# The Objects of Science

**Published:** 1909-01-30

**Authors:** 


### The Objects of Science.

"The proper study of mankind is man" is an aphorism which has of late years been over-interpreted or else under-estimated. On the one hand, we do not need to minimise the importance of the animal world as opposed to the civilised world, where during extensive periods of time man existed not upon the earth. There has been an age of reptiles, an age of birds, and the last and youngest predominant type is man. But though he be so young, he is the only creature that has been able to leave conscious records of his thoughts and actions for his posterity. To us, therefore, in whom has appeared this power of leaving records, the most interesting study should be man, for he is the creature we can best understand. But the recognition of man as a field for investigation brings with it the necessity of some basic principles which must guide our investigation. All scientific research into nature, whether it be botanical, chemical, physical, or zoological, must radiate to or from one centre—man. We are not concerned with the establishment of any new creed or perfervid altruism, but it seems not unreasonable to suppose that all our efforts should have one ultimate end, the amelioration of the conditions of life for our species and the development of an even higher type.

Scientific research which concerns itself only with minute observations is not and cannot be an end in itself. The man who for years studies the nervous system of some obscure crustacean, and thinks that at the end his object is gained, is a mere scientologist, one who talks about science but has no philosophical understanding. Such is the tendency on one side, where man is regarded as no more interesting than a group of lepidoptera or tunicata. On the other hand, we have the equally mistaken type of person who goes about disseminating the semi-complete theories of investigators without being able to show any original research or understanding on his own part. Breadth without depth is as fallacious as depth without breadth. Unfortunately the tendency of modern days is to advertise, and many a fine piece of potential work has been marred by a craze for notoriety. Nor are the sweeping generalisations which pass current now as the last word of wisdom to be regarded without suspicion. It is a popular form of error to discover violent antitheses that do not exist, but it does not help mankind in general.

The object of science must be primarily the advancement of man. We want the combination of the spirit of philosophy with the rigidity and exactitude of the pure scientist. Perhaps the finest example of such a combination was manifested by Herbert Spencer, whose true value as a thinker will not be grasped for some time. Historical perspective is needed; it takes several centuries to appreciate a Galileo or a Spinoza. Unless there be before each worker the final goal of higher advancement, his work is useful, but far less useful than it might have been. Why should we have expensive scientific education? Why give expression to and desire for this and that science if the end were the establishment of some side-issue, such, for example, as the inheritance of coat colour in mice? These points are studied only in so far as they will bear upon the upward path of man—if such a path be possible. Whether animals possess a psychical something, which for want of a better word we term soul, we do not know for certain; but it is quite evident that there is a psychical part in man. For this reason he has aspirations, and the handmaid of his soul is science.

